# Deep brain stimulation for chronic pain: a systematic review and meta-analysis

**DOI:** 10.3389/fnhum.2023.1297894

**Published:** 2023-11-30

**Authors:** Nour Shaheen, Ahmed Shaheen, Abdelrahman Elgendy, Yarema B. Bezchlibnyk, Theresa Zesiewicz, Brian Dalm, Jennifer Jain, Alexander L. Green, Tipu Z. Aziz, Oliver Flouty

**Affiliations:** ^1^Alexandria Faculty of Medicine, Alexandria, Egypt; ^2^Faculty of Medicine, Mansoura University, Mansoura, Egypt; ^3^Department of Neurosurgery and Brain Repair, University of South Florida, Tampa, FL, United States; ^4^Department of Neurology, University of South Florida, Tampa, FL, United States; ^5^Department of Neurosurgery, The Ohio State University, Columbus, OH, United States; ^6^Oxford Functional Neurosurgery, Department of Neurosurgery, John Radcliffe Hospital, University of Oxford, Oxford, United Kingdom

**Keywords:** chronic pain, pain, deep brain stimulation, DBS, neuromodulation, neurostimulation

## Abstract

**Background:**

Deep brain stimulation (DBS) has shown promise in effectively treating chronic pain. This study aimed to assess the efficacy of DBS in this context.

**Methods:**

We conducted a systematic literature search using PubMed, Scopus, and Web of Science, following the PRISMA guidelines. A well-constructed search strategy was utilized. Our literature search identified two groups of subjects: one group underwent DBS specifically for chronic pain treatment (DBS-P), while the second group received DBS for other indications (DBS-O), such as Parkinson’s disease or dystonia, with pain perception investigated as a secondary outcome in this population. Meta-analysis was performed using R version 4.2.3 software. Heterogeneity was assessed using the tau^2 and I^2 indices, and Cochran’s *Q*-test was conducted.

**Results:**

The analysis included 966 patients in 43 original research studies with chronic pain who underwent DBS (340 for DBS-P and 625 for DBS-O). Subgroup analysis revealed that DBS-P exhibited a significant effect on chronic pain relief, with a standardized mean difference (SMD) of 1.65 and a 95% confidence interval (CI) of [1.31; 2.00]. Significant heterogeneity was observed among the studies, with an I^2 value of 85.8%. However, no significant difference was found between DBS-P and DBS-O subgroups. Subgroup analyses based on study design, age, pain diseases, and brain targets demonstrated varying levels of evidence for the effectiveness of DBS across different subgroups. Additionally, meta-regression analyses showed no significant relationship between age or pain duration and DBS effectiveness for chronic pain.

**Conclusion:**

These findings significantly contribute to the expanding body of knowledge regarding the utility of DBS in the management of chronic pain. The study underscores the importance of conducting further research to enhance treatment outcomes and elucidate patient-specific factors that are associated with treatment response.

**Systematic review registration:**

https://www.crd.york.ac.uk/prospero/display_record.php?RecordID=428442, identifier CRD42023428442.

## 1 Introduction

Chronic pain refers to a persistent type of pain that extends beyond the expected duration for healing, commonly lasting for a period exceeding 3 months. This condition gives rise to a wide range of adverse effects, encompassing medical, social, and economic consequences. Individuals experiencing chronic pain may encounter challenges in their interpersonal relationships, witness a decline in productivity, and face escalated healthcare expenditures (Chou et al., 2015). According to the Institute of Medicine, pain constitutes a significant public health concern with profound financial implications for the United States. It has been estimated that the economic burden associated with pain, encompassing healthcare expenditures and diminished productivity, ranges from $560 to $635 billion annually (Institute of Medicine (US) Committee on Advancing Pain Research, Care, and Education, 2011). In recent years, studies have shown that in modern industrialized nations, prevalence studies indicate that approximately 20–30% of the population experience chronic pain across various conditions (Leadley et al., 2012; Karra et al., 2021). Given the prevalent occurrence and long-lasting nature of chronic pain, along with the undesirable consequences linked to dependence on pain medication, there has been an increasing emphasis on treatment approaches that integrate adjunctive therapies or non-pharmacological alternatives (Chiesa and Serretti, 2011).

Neurosurgeons commonly employ DBS as a therapeutic technique involving the implantation of electrodes in specific subcortical regions of the brain to administer electrical currents. The primary objective of this procedure is to address various movement disorders, notably Parkinson’s disease (PD), dystonia, and essential tremor (ET). However, scientists and medical professionals are actively investigating additional potential applications of DBS in the treatment of conditions such as obsessive-compulsive disorder, Tourette’s syndrome, depression, cluster headache (CH), and epilepsy. It is worth noting that the utilization of DBS for managing chronic pain dates back to the early 1970’s (Bittar et al., 2005a; Hamani et al., 2006).

Deep brain stimulation presents itself as an appealing option in the field of neurosurgery due to its minimally invasive nature, setting it apart from other surgical techniques. Additionally, DBS is generally well-tolerated by patients. In comparison to alternative neurosurgical methods, DBS carries a significantly lower risk. Notably, it falls under the category of neuromodulation, allowing for adjustments and reversibility, unlike previous procedures that involved the creation of brain lesions (Tan et al., 2023).

Two multicenter studies were conducted to assess the efficacy of DBS for pain relief and obtain approval from the FDA. However, both trials failed to meet the predetermined efficacy criteria, which required that a minimum of 50% pain relief be reported by at least half of the patients 1 year after the surgery. The pursuit of FDA approval was discontinued, and the utilization of DBS for pain management has been considered “off-label. Consequently, medical insurance reimbursement for this procedure is lacking. As a result, only a limited number of surgeons currently perform DBS for pain outside of Europe, where it has been approved by the European Federation of Neurological Societies (EFNS) and the United Kingdom National Institute for Health and Clinical Excellence (NICE) (Cruccu et al., 2007; National Institute for Health and Clinical Excellence, 2010).

Deep brain stimulation has shown efficacy in treating various pain syndromes, including post-stroke pain, phantom limb pain, brachial plexus injury, failed back surgery syndrome and the pain accompanying the PD (Bittar et al., 2005a; Falowski, 2015; Frizon et al., 2020; Flouty et al., 2022). Nevertheless, despite the available information, there continues to be a persistent drive to investigate and enhance the utility of DBS in the management of chronic pain. Our study aims to assess the impact of DBS on pain relief in two distinct contexts. We will first examine the effects of DBS specifically targeting chronic pain as the primary indication (DBS-P). Secondly, we will investigate the efficacy of DBS in alleviating pain when it is implanted for indications other than pain (DBS-O). By evaluating these different scenarios, we aim to gain a comprehensive understanding of the role of DBS in pain management across various conditions, contributing to the advancement of therapeutic interventions in the field.

## 2 Materials and methods

This study’s methodology adhered to the Cochrane Handbook for Systematic Reviews of Interventions (Higgins et al., 2023), incorporating guidelines for comprehensive literature searches, rigorous study selection criteria, and robust data synthesis techniques. Moreover, it followed the Preferred Reporting Items for Systematic Reviews and Meta-Analyses (PRISMA) guidelines (Page et al., 2021), ensuring transparent reporting of study selection, data extraction, and meta-analysis procedures according to Lee et al. (2022). The study protocol was prospectively registered in PROSPERO (CRD42023428442), ensuring transparency and adherence to established research guidelines.

In this meta-analysis, we adhered to the PICO framework for formulating our research question as outlined below:

**Population** (P): We focused on two distinct patient populations, categorized as follows:

- DBS-P: Patients with chronic pain as the primary indication for deep brain stimulation (DBS).
- DBS-O: Patients with indications other than chronic pain, such as Parkinson’s disease or dystonia, who underwent DBS.

**Intervention** (I): The primary intervention under examination was Deep Brain Stimulation (DBS).

**Comparison** (C): Our analysis encompassed several key comparative aspects:

- Evaluation of pain scores before DBS stimulation compared to pain scores after DBS stimulation (or ‘DBS off’).
- Assessment of the effectiveness of DBS in relation to movement disorders (e.g., Parkinson’s disease, dystonia).
- Investigation of the impact of stimulation targets (brain regions) on pain alleviation.
- Examination of the relationship between DBS and pain duration.

**Outcome** (O): Our primary outcome of interest was the quantification of pain reduction following DBS stimulation.

### 2.1 Search strategy

We searched the electronic databases (PubMed, Scopus, Web of science) using the following keywords: (“Chronic Pain” OR “Pain”[Mesh]) AND (“Deep Brain Stimulation” OR “DBS” OR “neuromodulation” OR “Neurostimulation”[Mesh]). The search was limited to articles published in English and published in peer-reviewed journals.

### 2.2 Eligibility criteria

The inclusion criteria for articles were that they were written in English, published in a peer-reviewed academic journal, and pertained to interventions or treatments, rehabilitation, or epidemiological examinations of pain. We excluded studies involving animals, reviews, descriptive articles, case reports that do not include the available outcomes, book chapters, and technical notes from the meta-analysis, evaluating them on an individual basis.

### 2.3 Data synthesis

We independently conducted the selection of articles, data extraction, and assessment of methodological quality. Outcome measures were extracted from diverse studies based on predefined inclusion criteria, following the quality assessment criteria outlined by the Oxford Centre for Evidence-Based Medicine levels of evidence (levels I-V) (Marx et al., 2015). Study characteristics were summarized, encompassing details such as DBS location, pain type, primary and secondary outcomes, DBS nucleus target subgroup, pain duration, duration subgrouping, pain questionnaire tools, pain scale utilized, pain relief achieved, effect size, standard deviation of the effect size, percentage of pain reduction, and follow-up duration in years. Treatment effect sizes were computed from reported means and variances of pre- and post-DBS data. Pain levels were assessed using standardized scales, including CH-QoL Pain scale, EUROQOL EQ-5D VAS, Kansas City Pain Disability Scale (KPDPS), McGill Pain Questionnaire (MPQ), among others (see Supplementary Table 1).

### 2.4 Statistical analysis

Statistical analysis was conducted in R using the meta package for a meta-analysis. Data, imported with readxl, underwent meta-analysis using the metacont function, specifying patient numbers, pre- and post-treatment means, standard deviations, and effect size (SMD). Forest plots were generated for visualization. Summary statistics, funnel plots, and sensitivity analyses were performed using various functions. The interpretation referred to Egger et al. (1997) BMJ study (Egger et al., 1997). To address pain measure heterogeneity, effect sizes were computed for each outcome, aggregated through averaging. Metacont function was reapplied for subgroup analysis based on study design, DBS brain target, or meta-regression on age and pain duration. Sensitivity analysis assessed the pooled estimate’s robustness by exploring individual studies or methodological choices.

## 3 Results

### 3.1 Literature search

A total of 1,106 records were obtained across all databases. After eliminating duplicated articles, 439 journal articles were selected based on their relevance to DBS as a treatment for addiction. Subsequently, these 439 articles underwent screening using our pre-assigned inclusion criteria, leading to the identification of 43 (21 for DBS-P and 16 for DBS-O) original research studies that met the potential eligibility requirements for inclusion in the study Supplementary Figure 1: PRISMA flow diagram.

### 3.2 Study characteristics

The analysis encompassed 966 patients with chronic pain who underwent DBS (340 for DBS-P and 625 for DBS-O), with an average age of 52.8 ± 11.2 years and a mean pain duration of 11.6 ± 10.3 years. The sample consisted of 40% females. The average follow-up period was 2.2 ± 2 years. The findings from the meta-analysis revealed that DBS exhibited a significant reduction in chronic pain, with an average pain reduction of 47.67 ± 20.01% for the DBS-P group and 59.59 ± 23.81% [51.01 ± 21.4% for both groups] (Table 1).

**TABLE 1:** Study design, countries, number of patients, patient demographics (age and sex), DBS location, the type of pain being treated, the reason for using DBS, pain duration, pain scale used, pain reduction percentage, the follow-up duration in years and quality assessment.

References	Study design	Country	Number of patients	Age (mean ± SD)	Sex-female (No)	DBS location	Pain	DBS-indication	DBS nucleus target subgroup	Pain duration	The pain scale used	Pain score improvement		Pain Reduction (%)	Follow-up duration (years)	Quality assessment
												Effect size	SD (effect size)			
For pain																
Saway et al., 2023	Case report	United States	1	59	0	VPM, MCS	Facial neuropathic pain	DBS-P		5	VAS	6	1	66.67	1	4
Abreu et al., 2022	Prospective	Portugal	16	53 ± 69.1	2	VPL	Post-traumatic neuropathic limb pain	DBS-P	VPL	20.2 ± 12.8	VAS	6.620847109	0.7551903733	76.9	5	1b
Krüger et al., 2021	RCT	Canada	1	63	1	CM, VPM, anterior pulvinar.	Neuropathic dental pain (NDP)	DBS-P			VAS	1.272792206	3.535533906	70	1	1b
Cappon et al., 2021	Clinical trial	United Kingdom	10	45.4 ± 11.9	2	VTA	Headache	DBS-P	VTA	16.0 ± 7.1	CH-QoL Pain	0.7175929249	1.254192968	11.8	1	3b
Kashanian et al., 2020	Case series	United States	9	57 ± 15.5	4	VPM, PVG	Facial Pain	DBS-P		6.8 ± 6	VAS	1.965795847	1.780449381	33	3.36 ± 4.4	4
Polanski et al., 2019	RCT	Germany	3	59.7 ± 3.9	1	PLIC, VPL, PVG	Chronic pain after brachial plexus injury	DBS-P		29 ± 9.9	NRS	4.880361175	0.6147086031	59.1	1	1b
Levi et al., 2019	Clinical trial	Italy	5	55.8 ± 8.96	1	Dorsal ACC	Thalamic pain syndrome (TPS)	DBS-P	ACC	5 ± 1.58	NRS	3.409584548	0.9385307667	35	1.5	3b
Cappon et al., 2019	Case series	United Kingdom	18	46.73	3	VTA	Chronic cluster headache	DBS-P	VTA	2	MPQ	−0.3593330256	11.74398037	−9.16	1.17 ± 0.38	4
Ben-Haim et al., 2018	Clinical trial	United States	7	55.1 ± 9.25	3	VPM, PAG	Neuropathic facial pain	DBS-P		14.4 ± 9.75	VAS	5.734172473	1.116115713	70	1	3b
Holland et al., 2018	Case report	United States	1	60	1	VC/VS, GPi	Entral post-stroke pain	DBS-P		7	MPQ	0.7	0	70.59	1	4
Abreu et al., 2017, 2022	Prospective	Portugal	16	53 ± 9.1	2	VPN thalamus	Neuropathic pain due to traumas	DBS-P		20.2 ± 2.8	VAS	3.106610192	1.36805062	53.1	3	1b
Lempka et al., 2017	RCT	United States	10	51.3 ± 4.75	4	VS/ALIC	Post-stroke pain	DBS-P		4.7 ± 2	VAS	−1.400619157	4.540848931	3.4	2	1b
Boccard et al., 2017	Clinical trial	United Kingdom	24	49.1 ± 11.2	5	ACC	Neuropathic Pain	DBS-P	ACC	NA	NRS	8.285651982	0.4706931945	47.56	3.2	3b
Kim et al., 2016	Clinical trial	United States	5	56 ± 14.8	3	VPN, VPL, PAG	Medically refractory pain	DBS-P		9.4 ± 9.5	NRS	16.90308509	0.2958039892	66.7	2.4 ± 0.96	3b
Rezaei Haddad et al., 2015	Case report	United States	1	50	0	VPL, VPM	body pain syndrome	DBS-P		7	OS	0.6	0	40	3.2	4
Son et al., 2014	Clinical trial	Republic of Korea	9	49.3 ± 10.75	3	contralateral VC thalamus	Chronic intractable neuropathic pain	DBS-P		8.1 ± 12.2	NRS	3.237075006	0.926762585	37.5	3.2 ± 1.9	3b
Gray et al., 2014	Prospective	United Kingdom	18	50.42 ± 10.37	4	PVG/PAG, ST	Neuropathic pain	DBS-P	PVG and PAG	7.25 ± 4.16	MPQ	1.296240837	10.41473129	42.267	0.5	1b
Pereira et al., 2013	Clinical trial	Portugal	12	53 ± 10	2	VPL	Neuropathic pain	DBS-P	VPL	20 ± 13	VAS	2.087614349	2.299275248	52.7	1	3b
Boccard et al., 2013	prospective cohort	United States	85	52.1 ± 13.3	25	PVG, VPL/VPM	Neuropathic pain	DBS-P		2	VAS	2.418052643	1.488801334	45.8	1.6	1b
Pereira et al., 2010	Clinical trial	United Kingdom	16	51 ± 14.3	3	PAG	Chronic neuropathic pain	DBS-P	PAG	9.44 ± 6.68	VAS	3.077891804	1.033174719	84	NA	3b
Owen et al., 2008	Clinical trial	United Kingdom	4	NA	NA	PVG and PAG	Chronic pain	DBS-P	PVG and PAG	NA	MPQ PRI	0.8809297784	18.67345208	53.5	NA	3b
Owen et al., 2007	Clinical trial	United Kingdom	34	50.4 ± 13	10	PVG, VPL/VPM	Neuropathic pain	DBS-P		NA	VAS	3.034738077	1.519076732	53.29	1.54 ± 0.9	3b
Pereira et al., 2007	Clinical trial	United Kingdom	3	52.7 ± 8.14	1	PVG and VPL	Chronic neuropathic pain	DBS-P	PVG and VPL	4.8	VAS	14.07232143	2.544001015	39.65	1	3b
Spooner et al., 2007	Case report	United States	1	40	0	PVG, cingulum	Neuropathic pain	DBS-P		12	VAS	19.79898987	0.1767766953	43.75	1	4
Owen et al., 2006	Clinical trial	United Kingdom	12	57.4 ± 10.8	3	PVG and VPL	Post-stroke pain	DBS-P	PVG and VPL	5.2	VAS	2.47042262	1.619156159	48.8	2.25	3b
Green et al., 2006	Prospective	United Kingdom	16	52	3	PAG	Chronic neuropathic pain	DBS-P	PAG	NA	MPQ	3.070536746	7.099735911	64.1	1	1b
Bittar et al., 2005b	Comparative Study	United Kingdom	3	55.67 ± 19.1	0	PVG, TS	Chronic neuropathic pain	DBS-P		NA	EUROQ OL EQ-5D VAS	1.984143634	11.79350104	61.7	1.12 ± 0.25	2a
For other conditions																
Wang et al., 2020	Retrospective	China	23	41.13 ± 13.49	10	GPi	Cervical DYT	DBS-O	GPi	3.6 ± 4.5	TWSTR S pain	2.203138278	2.555445592	71.7	1.59 ± 1.4	2b
Perides et al., 2020; Krüger et al., 2021	Retrospective	United Kingdom	138	11.5 ± 4	69	GPi	Dystonic pain	DBS-O	GPi	11.5 ± 4	NPRS	2.821223801	1.772280525	68.5	1	2b
Perides et al., 2020; Krüger et al., 2021	Retrospective	United Kingdom	2	11.5 ± 5	69	STN	Dystonic pain	DBS-O	STN	11.5 ± 5	NPRS	2.431801915	2.056088519	68.5	1	2b
Gong et al., 2020	Retrospective	China	36	62.3 ± 10.4	9	STN	PD-Related Pain	DBS-O	STN	3.3 ± 3.4	NRS	4.465178791	0.7054588735	79 ± 27	8	2b
Gong et al., 2020	Retrospective	China	28	63.2 ± 9.1	15	GPi	PD-Related Pain	DBS-O	GPi	2.1 ± 1.7	NRS	4.435433621	0.7890998488	75 ± 27		2b
Kaelin-Lang et al., 2020	Prospective	Switzerland	5	39 ± 6.75	3	GPi	Cervical DYT	DBS-O	GPi	5 ± 1.25	TWSTR S pain score	0.2931841924	4.43407262	16.5	11.5 ± 0.7	1b
DiMarzio et al., 2018	Prospective	United States	18	63.8 ± 8.5	4	STN, GPi	PD pain	DBS-O		11.6 ± 1.02	KPDPS	5.330780047	2.645016278	54.7	0.5	1b
Fabbri et al., 2017	Cross-sectional study	Portugal	32	62.5 ± 13.3	13	STN	PD-related pain	DBS-O	STN	18.7 ± 5.1	VAS-p	0.3338166355	1.797393947	35.3	4.6 ± 1.3	3b
Cury et al., 2016	Clinical Trial	Brazil	37	59 ± 10.8	12	STN	PD-related pain	DBS-O	STN	15 ± 7.2	VAS	2.419615423	17.85820986	72.5	1	3b
Jung et al., 2015	Clinical Trial	Republic of Korea	24	59.1 ± 7.6	9	STN	PD-related pain	DBS-O	STN	18.0 ± 3.8	Ordinal scale from 0 to 1	0.899854933	1.555806329	22.58	8	3b
Sürücü et al., 2013	Retrospective	Switzerland	14	62.8 ± 5.69	6	STN	PD-related pain	DBS-O	STN	12.3 ± 3.82	Ordinal scale	2.304728268	2.451482059	65.9	1.3 ± 1	2b
Dellapina et al., 2012	RCT	France	8	65.1 ± 5.0	8	STN	PD-related pain	DBS-O	STN	12.4 ± 2.6	VAS	1.317009216	1.594521872	28	0.25	1b
Kim et al., 2012	Clinical Trial	Republic of Korea	21	58.3 ± 7.9	13	STN	PD-related pain	DBS-O	STN	10.6 ± 4.0	OS	1.424182765	1.123451315	23.53	2	3b
Oshima et al., 2012	Prospective	Japan	69	63.0 ± 7.8		STN	PD-related pain	DBS-O	STN	63.0 ± 7.8	VAS	3.90472786	1.075619134	80.77	1	1b
Cury et al., 2014	Clinical Trial	Brazil	44	60 ± 10.4	14	STN	PD-related pain	DBS-O	STN	6.52 ± 6.50	VAS	2.077258568	1.396070785	44.6		3b
Pellaprat et al., 2014	Prospective	France	58	60.3 ± 7.8	21	STN	PD-related pain	DBS-O	STN	12.3 ± 3.8	MPQ-QDSA	1.242343385	4.990568691	45	1	1b
Kim et al., 2008	Retrospective	Republic of Korea	29	59 ± 7.7	15	STN	PD-related pain	DBS-O	STN	9.9 ± 4.6	NPRS	1.021763182	1.174440439	19	0.25–0.5	2b
Witjas et al., 2007	Prospective	France	40	59 ± 8	10	STN	PD-related pain	DBS-O	STN	12.4 ± 4.5	NMF	3.866208623	0.3621118612	84.2	1	1b

### 3.3 DBS indication

The results of the subgroup analysis for DBS specifically targeting chronic pain (DBS-P) are as follows: In the random effects model, the standardized mean difference (SMD) for DBS-P is 1.65, with a 95% confidence interval (CI) of [1.31; 2.00]. The *z*-value is 9.45, and the corresponding *p*-value is less than 0.0001, indicating a significant effect of DBS-P on chronic pain.

Quantifying heterogeneity, the estimated tau^2 is 0.92, with a 95% CI of [0.61; 2.39]. The corresponding tau value is 0.9620, with a 95% CI of [0.78; 1.54]. The I^2 value, representing the percentage of total variation across studies due to heterogeneity, is 85.8%, with a 95% CI of [81.6%; 89.1%]. The estimated H value, which represents the ratio of total variation to sampling variation, is 2.66, with a 95% CI of [2.33; 3.03]. The test of heterogeneity shows a *Q*-value of 275.02 with 39 degrees of freedom and a *p*-value less than 0.0001, indicating significant heterogeneity among the studies.

Analyzing the subgroups within the random effects model, the DBS-P subgroup includes 22 studies. The SMD for DBS-P is 1.91, with a 95% CI of [1.32; 2.49]. The estimated tau^2 for this subgroup is 1.46, and the corresponding tau value is 1.2105. The *Q*-value for this subgroup is 134.93, with an I^2 value of 84.4%.

The DBS-O subgroup, which represents a different indication for DBS, includes 18 studies. The SMD for DBS-O is 1.4689, with a 95% CI of [1.07; 1.86]. The estimated tau^2 for this subgroup is 0.5747, and the corresponding tau value is 0.7581. The *Q*-value for this subgroup is 137.18, with an I^2 value of 87.6%.

**FIGURE 1:**
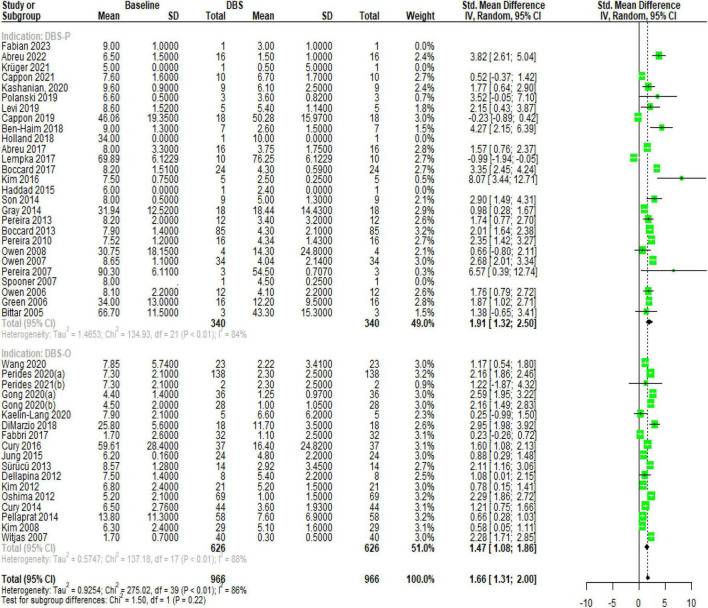
Forest plot, illustrating the significant effect of DBS on chronic pain and the substantial heterogeneity observed among the included studies.

The test for subgroup differences within the random effects model yields a *Q*-value of 1.50 with 1 degree of freedom and a *p*-value of 0.2213, indicating no significant difference between the DBS-P and DBS-O subgroups. Overall, these results suggest a significant positive effect of DBS specifically targeting chronic pain (DBS-P) based on the random effects model. However, there is significant heterogeneity among the studies, and no significant difference is observed between the DBS-P and DBS-O subgroups Figure 1.

### 3.4 Risk of bias

These results indicate that the linear regression test did not find significant asymmetry in the funnel plot. The test result shows a *t*-value of 0.82 with 38 degrees of freedom, resulting in a *p*-value of 0.41, which is greater than the conventional threshold for statistical significance (usually 0.05). The sample estimates provide additional information. The estimated bias is 0.71, with a standard error of 0.87. The intercept is estimated to be 1.33, with a standard error of 0.30.

Furthermore, the details of the analysis reveal the multiplicative residual heterogeneity variance, tau^2, which is calculated to be 7.11. Overall, these results suggest that there is no significant funnel plot asymmetry, indicating that publication bias or other forms of bias may not be influencing the results of the study (Supplementary Figure 2).

### 3.5 Sensitivity analysis

The sensitivity analysis explored the influence of omitting individual studies on the results. Here are the key findings: When omitting Saway et al. (2023), the SMD remained significant and had a similar effect size as the pooled estimate. The *p*-value remained <0.0001, indicating a significant effect of DBS on chronic pain relief. The heterogeneity measures (tau^2, tau, I^2) also remained similar. Similarly, omitting Holland et al. (2018), Cappon et al. (2021), Krüger et al. (2021), and Abreu et al. (2022) did not substantially affect the overall results. The *p*-value remained <0.0001, and the effect sizes and heterogeneity measures remained consistent. Omitting other studies also showed similar patterns. The *p*-value remained <0.0001, and the effect sizes (SMD) and heterogeneity measures (tau^2, tau, I^2) were relatively stable. The pooled estimate, representing the overall effect of DBS on chronic pain relief, remained statistically significant with a *p*-value <0.0001. The SMD, 95% CI, and heterogeneity measures (tau^2, tau, I^2) were consistent with the original analysis. Overall, the sensitivity analysis suggests that the findings of the study are robust. Omitting individual studies did not significantly alter the overall results or conclusions regarding the effectiveness of DBS for chronic pain relief (Supplementary Figure 3).

## 4 Subgroup analysis

### 4.1 Study designs

The subgroup analysis of DBS specifically targeting chronic pain (DBS-P) revealed different subgroups based on study design. Among the subgroups, the observational studies-P subgroup (2 studies) showed an SMD of 0.71 with a 95% CI of [−1.24; 2.67], indicating a moderate effect size. The cohort-P subgroup (6 studies) demonstrated a higher SMD of 1.9087 with a narrower CI of [1.18; 2.63]. The RCT-P subgroup (2 studies) displayed a lower SMD of 0.92 with a wide CI of [−3.45; 5.29], suggesting substantial heterogeneity. The non-RCT-P subgroup (12 studies) had the largest effect size of 2.3298 and a relatively narrow CI of [1.63; 3.02]. The cohort-O subgroup (12 studies) had an SMD of 1.74 and a CI of [1.24; 2.24]. The remaining subgroups, observational studies-O, non-RCT-O, RCT-O, and non-RCT, each had only one study, and their effect sizes ranged from 0.23 to 1.20. The tests for subgroup differences indicated significant heterogeneity among the subgroups (*Q* = 33.41, df = 8, *p* < 0.0001), suggesting that the effectiveness of DBS for chronic pain varied across different study designs (Supplementary Figure 4).

### 4.2 Pain diseases

The results suggest varying levels of evidence for the effectiveness of DBS across different pain subgroups. The subgroup of facial neuropathic pain (P) and traumatic pain (P) showed strong evidence for a significant effect, with SMDs of 2.85 (95% CI: 0.43 to 5.27) with a moderate tau^2 value of 2.35 and substantial heterogeneity (I2 = 75.8%), and 2.77 (95% CI: 1.05 to 4.48) with tau^2 = 1.55 and I^2 = 79.3%, respectively. The subgroup of chronic cluster headache (P) presented weak evidence, with an SMD of 0.0809 (95% CI: −0.64 to 0.80) with low heterogeneity (tau^2 = 0.1246, I^2 = 43.8%). The subgroups of post-stroke pain (P), dystonic pain (O), and PD-related pain (O) exhibited moderate evidence, with SMDs of 0.37 (95% CI: −2.31 to 3.07) with higher heterogeneity (tau^2 = 3.5450, I^2 = 93.7%), 1.33 (95% CI: 0.40 to 2.26) with tau^2 = 0.58 and I^2 = 79.9%, and 1.50 (95% CI: 1.05 to 1.94) with tau^2 = 0.62 and I^2 = 88.2%, respectively. The test for subgroup differences was statistically significant (*Q* = 16.18, df = 5, *p* = 0.0063), indicating that the effectiveness of DBS varies among different pain subgroups (Supplementary Figure 5).

### 4.3 Brain targets

Deep brain stimulation primary for treating chronic pain (DBS-P) analysis: The subgroup analysis revealed variations in the effect sizes of DBS targeting different brain nuclei for the treatment of chronic pain. The subgroup with the largest effect size was the ventral posterior lateral (VPL) thalamus, with an SMD of 2.74 (95% CI [0.70; 4.78]) with high heterogeneity (tau^2 = 1.86, I^2 = 85.7%), indicating a large treatment effect. The subgroups of anterior cingulate cortex (ACC) and PAG also showed significant effects, with SMDs of 2.98 (95% CI [1.90; 4.06]) with low heterogeneity (tau^2 = 0.2265, I^2 = 31.7%), and 2.08 (95% CI [1.46; 2.71]) with no observed heterogeneity, respectively, suggesting moderate to large treatment effects. On the other hand, the ventral tegmental area (VTA) and periaqueductal/periventricular gray matter region (PVG and PAG) subgroups demonstrated small and non-significant effects, with SMDs of 0.0809 (95% CI [−0.64; 0.80]) with low heterogeneity (tau^2 = 0.12, I^2 = 43.8%) and 0.9176 (95% CI [0.29; 1.54]) with no observed heterogeneity, respectively. The subgroup of PVG and VPL had a wide confidence interval and substantial heterogeneity (tau^2 = 6.49, I^2 = 56.1%), making the treatment effect uncertain.

**FIGURE 2:**
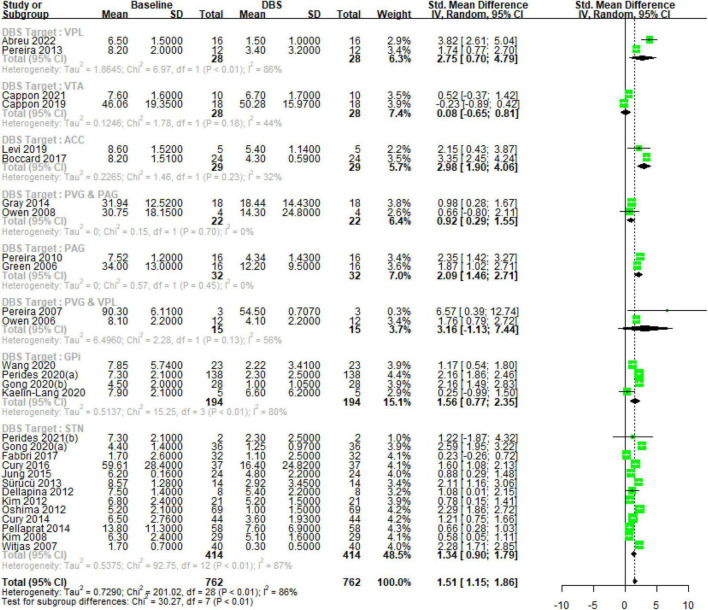
Deep brain stimulation (DBS) brain target nuclei subgroup analysis.

Deep brain stimulation for other indications (DBS-O) analysis: The globus pallidus internus (GPi) subgroup exhibited a moderate effect size (SMD = 1.56, 95% CI [0.77; 2.35]) with high heterogeneity (tau^2 = 0.51, I^2 = 80.3%). The largest subgroup, subthalamic nucleus (STN), demonstrated a moderate effect size (SMD = 1.3425, 95% CI [0.89; 1.78]) with high heterogeneity (tau^2 = 0.53, I^2 = 87.1%). The test for subgroup differences indicated significant heterogeneity between the subgroups (*Q* = 30.27, df = 7, *p* < 0.0001), suggesting that the treatment effects varied significantly among the different DBS nucleus target subgroups Figure 2.

### 4.4 Meta-regression analysis

#### 4.4.1 Age meta-regression

The meta-regression analysis examined the relationship between age and the effectiveness of DBS for treating chronic pain. The analysis included three subgroups: overall (*k* = 42), DBS primary for treating chronic pain (DBS-P) (*k* = 24), and DBS for other indications (DBS-O) (*k* = 18), F (df1 = 1, df2 = 40) = 0.13, *p*-val = 0.71.

##### 4.4.1.1 Overall analysis of age meta-regression

The test of moderators for age as a predictor was not significant (*p* = 0.71), suggesting that age did not significantly moderate the effectiveness of DBS for chronic pain. The estimated amount of residual heterogeneity (tau^2) was 23.45, indicating significant heterogeneity among the studies. The I^2 value was 99.78%, indicating that most of the variability in the effect sizes was due to heterogeneity. The test for residual heterogeneity was highly significant (*p* < 0.0001).

##### 4.4.1.2 DBS primary for treating chronic pain (DBS-P) analysis

The test of moderators for age as a predictor was not significant F(df1 = 1, df2 = 22) = 1.1608, (*p* = 0.29), indicating that age did not significantly moderate the effectiveness of DBS-P for chronic pain The estimated amount of residual heterogeneity (tau^2) was 43.86, indicating considerable heterogeneity among the studies. The I^2 value was 99.81%, indicating a high proportion of variability in effect sizes due to heterogeneity. The test for residual heterogeneity was significant (*p* < 0.0001).

##### 4.4.1.3 DBS for other indications (DBS-O) analysis

**FIGURE 3:**
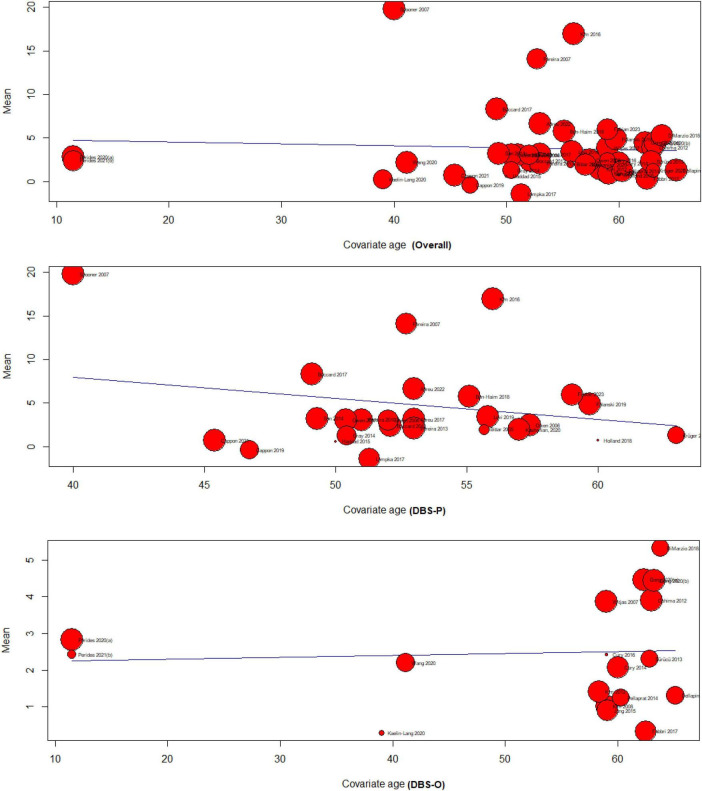
Age meta-regression analysis.

The test of moderators for age as a predictor was not significant F(df1 = 1, df2 = 16) = 0.04, (*p* = 0.83), indicating that age The estimated amount of residual heterogeneity (tau^2) was 1.46, indicating some residual heterogeneity among the studies. The I^2 value was 97.33%, suggesting a substantial proportion of variability in effect sizes due to heterogeneity. The test for residual heterogeneity was highly significant (*p* < 0.0001). did not significantly moderate the effectiveness of DBS-O for chronic pain Figure 3.

#### 4.4.2 Pain duration meta-regression

**FIGURE 4:**
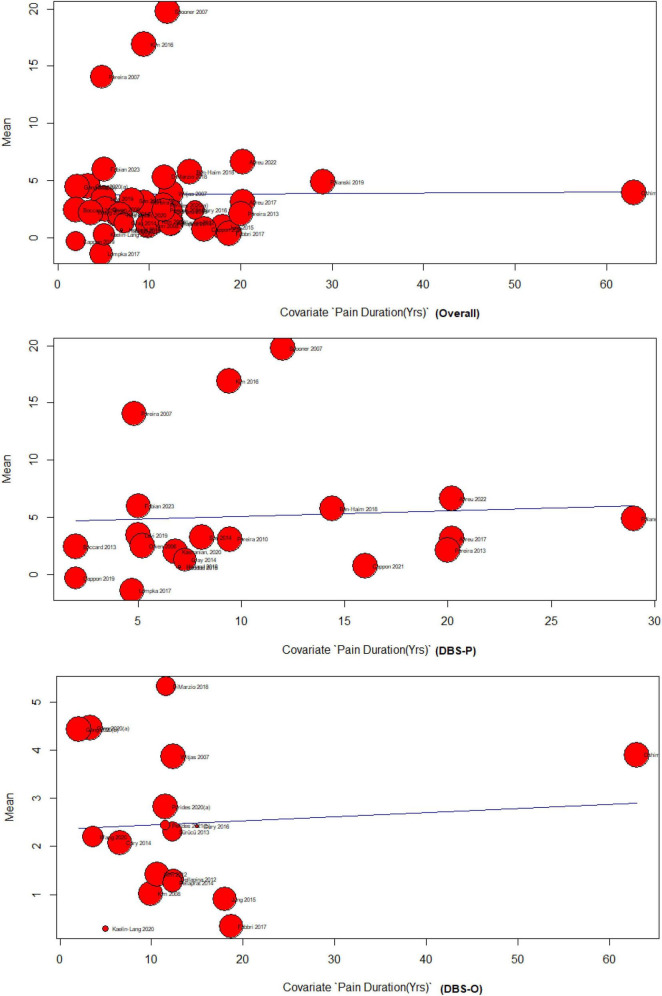
Pain duration meta-regression analysis.

The results of the meta-regression analyzing the effect of pain duration on DBS for treating chronic pain indicate limited evidence of a significant association. The overall analysis, including 37 studies, showed no significant relationship between pain duration and DBS effectiveness F (df1 = 1, df2 = 35) = 0.0028 (*p* = 0.95). The DBS primary subgroup analysis, comprising 19 studies, also found no significant association F(df1 = 1, df2 = 17) = 0.07 (*p* = 0.79), The DBS-O subgroup, consisting of 18 studies, demonstrated a weak but significant positive association between pain duration and DBS effectiveness (*p* = 0.74), F(df1 = 1, df2 = 16) = 0.10. However, it should be noted that the amount of heterogeneity accounted for was minimal across all analyses, indicating that pain duration explains only a small proportion of the variability in DBS outcomes for chronic pain Figure 4.

## 5 Discussion

### 5.1 Summary of findings

The current literature does not provide a clear consensus on the optimal use and effectiveness of DBS for chronic pain. This meta-analysis represents the first comprehensive investigation into the effects of DBS on chronic pain. To the best of our knowledge, no prior meta-analyses have examined the impact of DBS on chronic pain, whether it was utilized as a primary treatment modality or in conjunction with DBS for other medical conditions. The results revealed a significant effect of DBS-P on chronic pain, with SMD of 1.65 and a 95% CI of [1.31; 2.00]. However, notable heterogeneity among the studies was observed, as reflected by high I^2 values and significant *Q*-values. This heterogeneity can be attributed to the diverse brain targets selected for DBS application, as evident from the subgroup analysis based on brain target grouping.

Subgroup analyses based on study designs exhibited different effect sizes for DBS-P. Observational studies-P demonstrated a moderate effect size, while cohort-P and non-randomized controlled trials (non-RCT)-P subgroups showed larger effect sizes. The randomized controlled trials (RCT)-P subgroup exhibited substantial heterogeneity. The observed differences in effect sizes between RCTs and observational or cohort studies highlight the importance of study design in accurately capturing the effectiveness of DBS for chronic pain. This suggests that the more rigorous design of RCTs may provide a more accurate representation of DBS effectiveness. The marked difference in effect sizes between RCTs and non-RCTs underscores the potential limitations of non-randomized studies in truly representing the effect. It is imperative for readers and clinicians to approach the findings of non-RCTs with caution, bearing in mind the potential biases and confounders that might inflate or diminish the observed effects.

In the meta-regression analysis, the relationship between age and the effectiveness of DBS for chronic pain was explored. The results suggest that age is not a significant factor in determining the effectiveness of DBS for treating chronic pain in both DBS-P and DBS-O analyses. Also, the results show that DBS can be an effective treatment for some types of chronic pain that are resistant to other therapies. The meta-analysis also assessed the effectiveness of DBS across different pain subgroups. Facial neuropathic pain and traumatic pain showed strong evidence of a significant effect, while chronic cluster headache presented weak evidence. Post-stroke pain, dystonic pain, and PD-related pain demonstrated moderate evidence.

Regarding the meta-regression analyzing the effect of pain duration on DBS for chronic pain, limited evidence of a significant association was found. The overall analysis and the DBS-P subgroup analysis did not find a significant relationship, while the DBS-O subgroup showed a weak but significant positive association. Our study adds to the current literature complexity by revealing that pain duration may not reliably predict DBS outcomes. In our meta-regression analysis, we investigated the impact of pain duration on the effectiveness of DBS in treating chronic pain. However, the results indicate a scarcity of substantial evidence supporting a significant association between these variables. Both the overall analysis and the primary subgroup analysis focusing on DBS revealed no significant relationship between pain duration and DBS effectiveness (*p* = 0.95 and *p* = 0.79, respectively). Nonetheless, in the DBS-O subgroup analysis, we observed a weak but significant positive association between pain duration and DBS effectiveness (*p* = 0.74); however, this association only accounted for a small proportion of the variability in DBS outcomes.

The findings demonstrate that DBS applied to various brain nuclei has differing effects on chronic pain. Notably, the VPL, ACC, and PVG and PAG emerged as the most effective targets, exhibiting substantial or moderate treatment effects with significant SMD between the DBS and control groups. These targets are implicated in both the sensory-discriminative and affective components of pain perception (Frizon et al., 2020; Li et al., 2020). Conversely, the VTA and the combination of PVG and PAG displayed limited effectiveness, with small or non-significant effects. The VTA, being part of the reward system, may not directly contribute to pain modulation (Li et al., 2020). Furthermore, the combined stimulation of PVG and PAG may not be optimal due to the distinct roles these regions play in pain processing, potentially necessitating different stimulation parameters (Frizon et al., 2020). Regarding other targets, such as GPi, STN, and PVG and VPL combination, moderate effects were observed; however, high heterogeneity and wide confidence intervals indicate variability and uncertainty in treatment outcomes. These targets primarily participate in motor control and may exert indirect influences on pain by modulating movement disorders like dystonia (Rodrigues et al., 2019; Frizon et al., 2020; Fan et al., 2021).

DBS has been used for the treatment of chronic pain since the early 1970s, but it remains off-label in the United States and its indications are contested (Falowski, 2015). This technique has the capacity to modulate the activity of neural circuits associated with pain processing and perception (Falowski, 2015; Prosky et al., 2021; Alamri and Pereira, 2022). Although the precise mechanism underlying the pain-relieving effects of DBS remains incompletely understood, it is believed to involve various factors. These include the alteration of the balance between inhibitory and excitatory neurotransmitters within pain pathways, the reduction of activity in nociceptive signal-transmitting neurons, the enhancement of endogenous opioid systems responsible for mediating analgesia (Prosky et al., 2021; Alamri and Pereira, 2022), and the modification of emotional and cognitive aspects of pain such as anxiety, depression, and catastrophizing (Falowski, 2015; Prosky et al., 2021; Alamri and Pereira, 2022). The effects of DBS on chronic pain relief may vary depending on the specific brain target utilized. Some common targets include the sensory thalamus (ST), (ventral posterior lateral and ventral posterior medial), which is primarily associated with sensory-discriminative pain aspects such as location, intensity, and quality (Falowski, 2015; Prosky et al., 2021; Alamri and Pereira, 2022). the periaqueductal gray and periventricular gray matter, involved in descending pain modulation and endogenous opioid release; and the ACC, implicated in affective-motivational pain aspects such as unpleasantness, suffering, and coping (Falowski, 2015; Prosky et al., 2021; Alamri and Pereira, 2022).

### 5.2 Meta-analysis limitations

This meta-analysis represents the first comprehensive examination of deep brain stimulation (DBS) effectiveness in treating chronic pain. It encompasses various indications for DBS use, brain targets for stimulation, and potentially influential factors such as patient age and pain duration.

However, it is important to acknowledge the limitations of this study. Firstly, significant heterogeneity was observed among the included studies, as indicated by high I^2 values. This heterogeneity may arise from variations in patient characteristics, study designs, DBS techniques, and outcome measures employed across different studies. The presence of heterogeneity may restrict the generalizability of the findings and limit the ability to draw definitive conclusions. Secondly, due to ethical considerations, there was a lack of control group standardization. Implementing a sham or controlled procedure in patients who are already experiencing illness presents ethical challenges. As a result, it was not feasible to establish a standardized control group for comparison. This limitation needs to be taken into account when interpreting the study’s results. Hence, the study emphasizes the need for additional research especially RCTs to improve treatment results and better understand the patient-specific factors linked to treatment response.

## 6 Conclusion

The meta-analysis reveals a significant positive effect of DBS in reducing chronic pain. Subgroup analysis indicates a larger effect size in the DBS-P group compared to DBS-O, with varying effects based on study design, showing the most substantial effect in the non-RCT-P subgroup. Age did not significantly moderate the effectiveness of DBS. Strong evidence supports DBS effectiveness in facial neuropathic pain and traumatic pain subgroups, while weak evidence is found for chronic cluster headache. Pain duration did not significantly impact DBS effectiveness. VPL demonstrated the largest effect among different brain targets, with significant heterogeneity observed. These findings contribute valuable insights into DBS’s utility for chronic pain, emphasizing the need for further research to optimize outcomes and identify patient-specific factors influencing treatment response.

## Data availability statement

The original contributions presented in the study are included in the article/Supplementary material, further inquiries can be directed to the corresponding author.

## Author contributions

NS: Conceptualization, Data curation, Formal analysis, Methodology, Writing – original draft, Writing – review and editing. AS: Writing – review and editing, AE: Data curation, Writing – review and editing. YB: Writing – review and editing. TZ: Writing – review and editing. BD: Writing – review and editing. JJ: Writing – review and editing. AG: Writing – review and editing. TA: Writing – review and editing. OF: Conceptualization, Funding acquisition, Investigation, Methodology, Supervision, Writing – original draft, Writing – review and editing.
