# Supplementary material for: Deep brain stimulation for chronic pain: a systematic review and meta-analysis
Source: Front Hum Neurosci. 2023 Nov 30;17:1297894. doi: 10.3389/fnhum.2023.1297894 (PMC10719838; doi:10.3389/fnhum.2023.1297894)
Supplement: Supplementary file 1 [file Data_Sheet_1.docx]

**Identification of studies via databases and registers**

Records identified from: Total:**(n = 1,106)**

PubMed: (n = 327)

Scopus: (n = 436)

Web of science: (n =343)

Duplicate records removed:

(n =667)

**Identification**

Records excluded:(n = 184)

Records screened:

**(n =439)**

Reports not retrieved:

(n =182)

Editorials, Reviews, or Animal studies

Reports sought for retrieval:

**(n =255)**

**Screening**

Reports excluded (n =30)

Did not include outcomes according to our inclusion criteria.

Reports assessed for eligibility:

**(n =73)**

Studies included in review:

**(n =43)**

Reports of included studies:

**(n =43)**

**Included**

Figure 1S. PRISMA flow chart of the included studies in the systematic review.

*
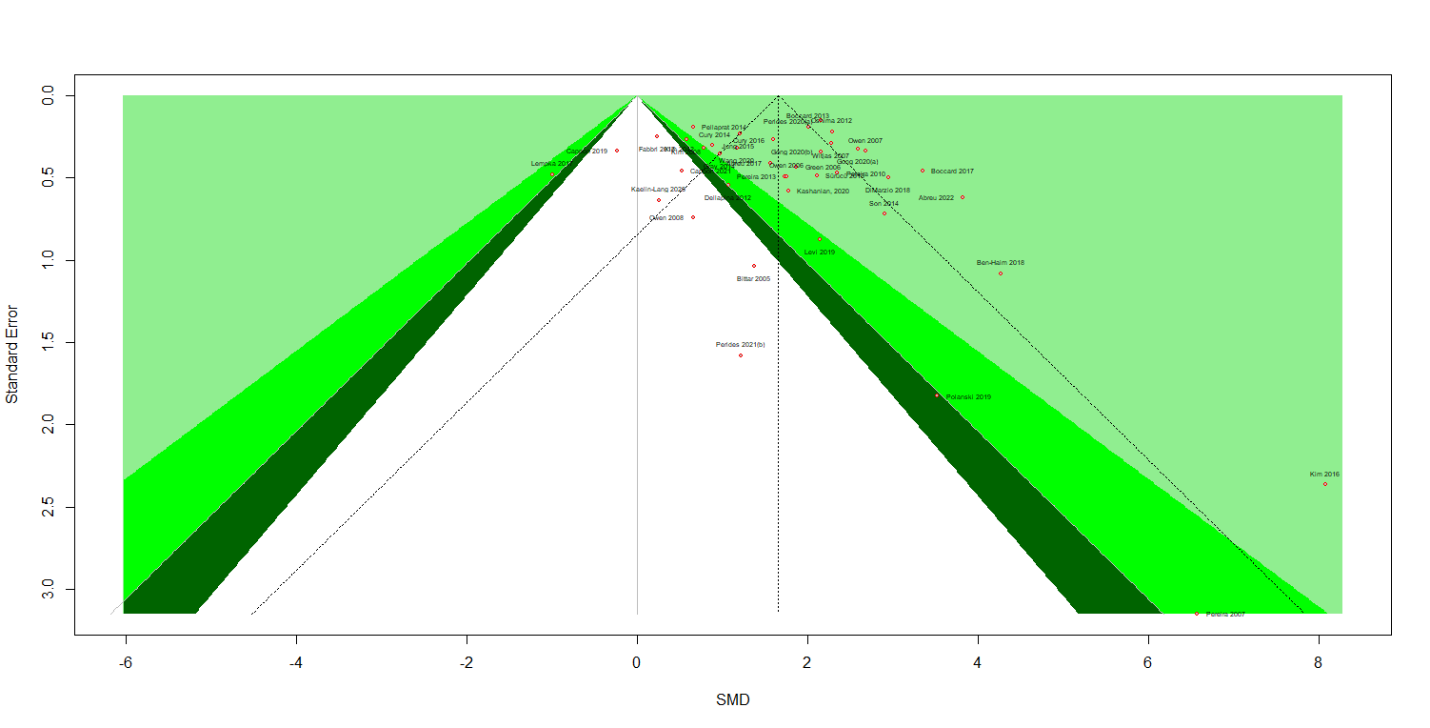
*

Figure 2S. funnel plot including studies of DBS applied for the management of chronic pain.


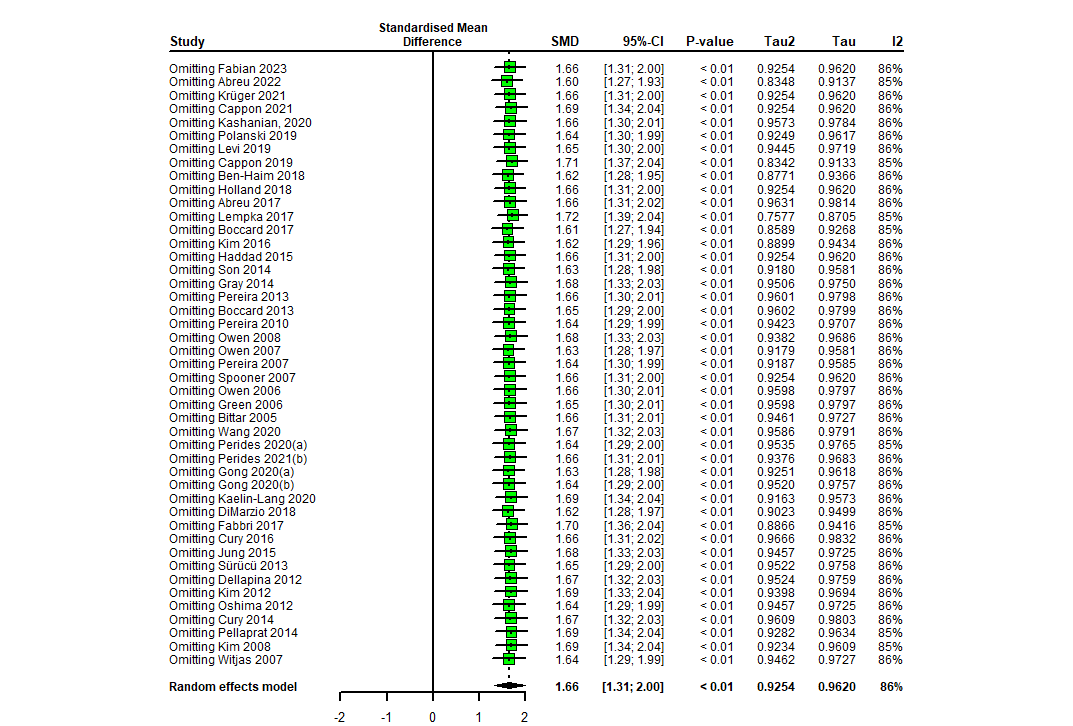


Figure 3S: Sensitivity Analysis showing impact of omitting various studies on the overall pooled estimate.


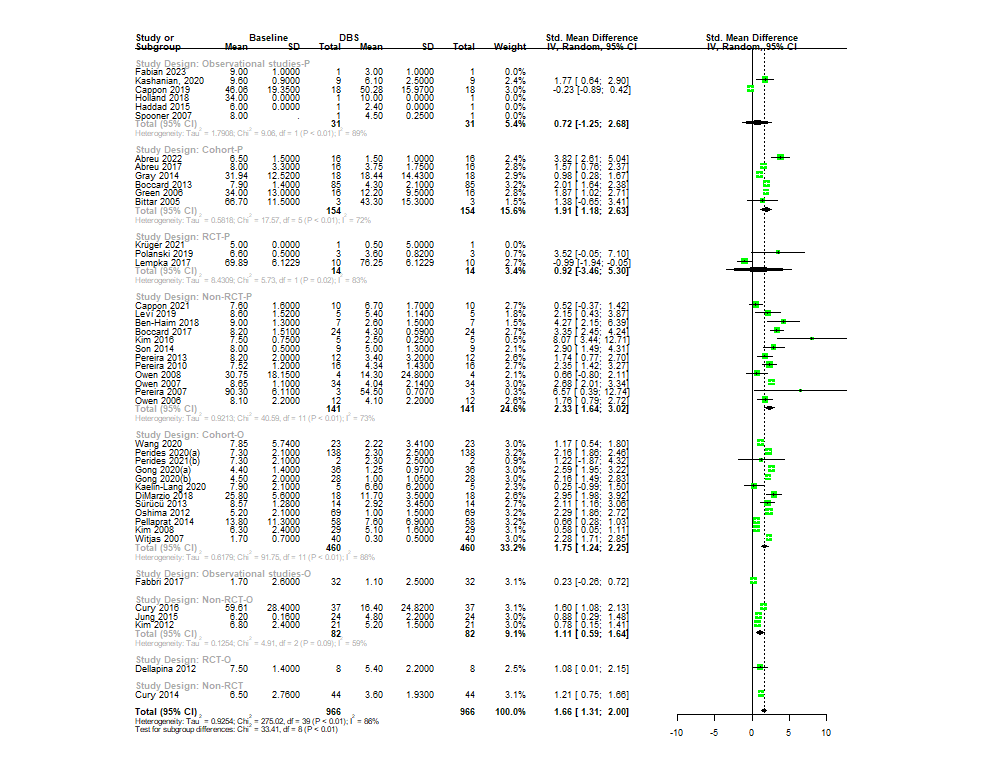


Figure 4S: Study Design Subgroup Analysis


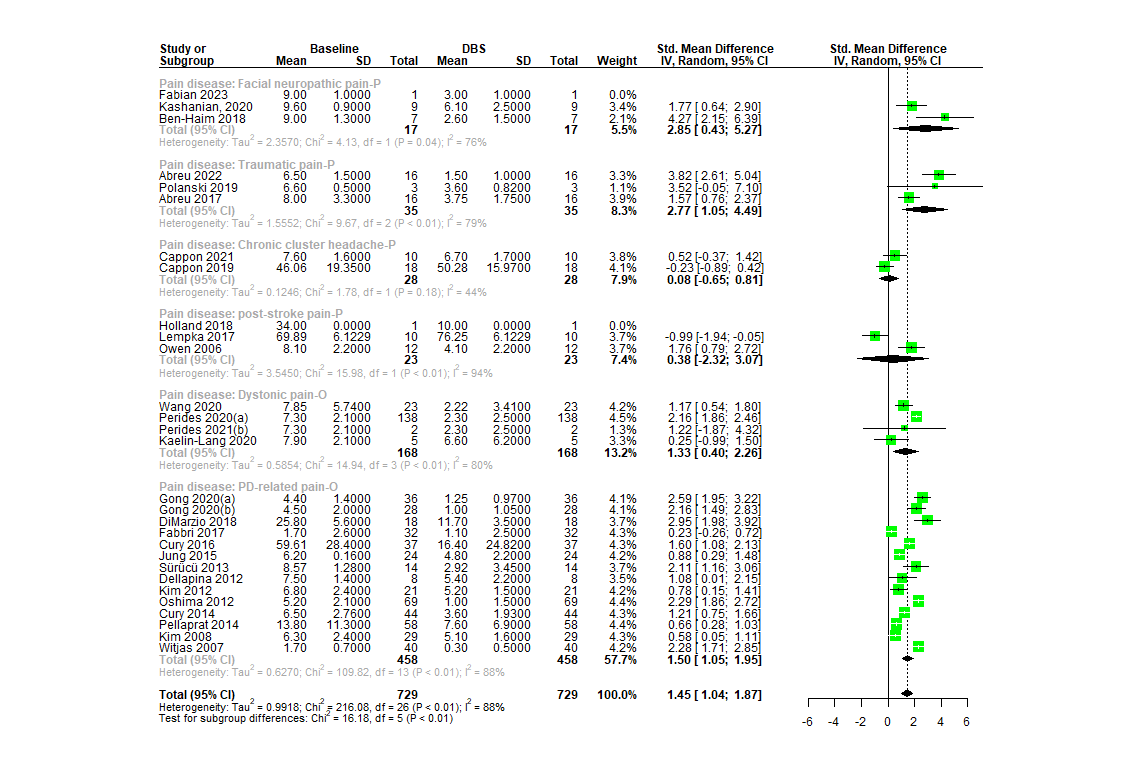


Figure 5S. Forest plot of different conditions of pain Subgroup analysis

**Table S1**. Search strategy for each database.

| **Database** | **Search query** | **Results** |
| --- | --- | --- |
| PubMed | (("Chronic Pain"[Mesh] OR "Pain"[Mesh]) AND ("Deep Brain Stimulation"[Mesh]) OR "neuromodulation"[Mesh] OR "Neurostimulation"[Mesh] OR “DBS” [Mesh]) | 327 |
| Scopus | TITLE-ABS-KEY ((“Chronic pain" OR "pain”) AND (“Deep Brain Stimulation" OR "neuromodulation" OR "Neurostimulation" OR “DBS”)) | 436 |
| Web of Science | ALL= ((“Chronic pain” OR “pain”) AND ("Deep Brain Stimulation" OR "neuromodulation" OR "Neurostimulation" OR “DBS”)) | 343 |
| Total: **1,106** | | |

| Pain Scale |
| --- |
| CH-QoL Pain scale |
| EUROQOL EQ-5D VAS |
| Kansas City Pain Disability Scale (KPDPS) |
| McGill Pain Questionnaire (MPQ) |
| McGill Pain Questionnaire - Short Form (MPQ-QDSA) |
| McGill Pain Rating Index (MPQ PRI) |
| Neuropathic Pain Diagnostic Questionnaire (NMF) |
| Numeric Pain Rating Scale (NPRS) |
| Numeric Rating Scale (NRS) |
| Ordinal Scale (OS) |
| Toronto Western Spasmodic Torticollis Rating Scale(TWSTRS) |
| VAS/VAS-P |

(Table for scales for the supplementary material)
